# Microbial Communities of Meat and Meat Products: An Exploratory Analysis of the Product Quality and Safety at Selected Enterprises in South Africa

**DOI:** 10.3390/microorganisms9030507

**Published:** 2021-02-27

**Authors:** Evelyn Madoroba, Kudakwashe Magwedere, Nyaradzo Stella Chaora, Itumeleng Matle, Farai Muchadeyi, Masenyabu Aletta Mathole, Rian Pierneef

**Affiliations:** 1 Department of Biochemistry and Microbiology, Faculty of Science and Agriculture, University of Zululand, KwaDlangezwa 3886, South Africa; 2 Directorate of Veterinary Public Health, Department of Agriculture, Land Reform and Rural Development, Pretoria 0001, South Africa; KudakwasheM@dalrrd.gov.za; 3 Department of Life and Consumer Sciences, College of Agriculture and Environmental Sciences, University of South Africa, Florida 1710, South Africa; nyarry.stch@gmail.com; 4 Biotechnology Platform, Agricultural Research Council, Private Bag X 05, Onderstepoort, Pretoria 0110, South Africa; MuchadeyiF@arc.agric.za (F.M.); PierneefR@arc.agric.za (R.P.); 5 Bacteriology Division, Agricultural Research Council, Onderstepoort Veterinary Research, Onderstepoort 0110, South Africa; MatleI@arc.agric.za (I.M.); MatholeM@arc.agric.za (M.A.M.)

**Keywords:** pathogens, one health, food safety, biosecurity, meat value chain, culture dependent techniques, culture-independent techniques, metagenomics

## Abstract

Consumption of food that is contaminated by microorganisms, chemicals, and toxins may lead to significant morbidity and mortality, which has negative socioeconomic and public health implications. Monitoring and surveillance of microbial diversity along the food value chain is a key component for hazard identification and evaluation of potential pathogen risks from farm to the consumer. The aim of this study was to determine the microbial diversity in meat and meat products from different enterprises and meat types in South Africa. Samples (*n* = 2017) were analyzed for *Yersinia enterocolitica*, *Salmonella* species, *Listeria monocytogenes*, *Campylobacter jejuni*, *Campylobacter coli*, *Staphylococcus aureus*, *Clostridium perfringens*, *Bacillus cereus*, and *Clostridium botulinum* using culture-based methods. PCR was used for confirmation of selected pathogens. Of the 2017 samples analyzed, microbial ecology was assessed for selected subsamples where next generation sequencing had been conducted, followed by the application of computational methods to reconstruct individual genomes from the respective sample (metagenomics). With the exception of *Clostridium botulinum*, selective culture-dependent methods revealed that samples were contaminated with at least one of the tested foodborne pathogens. The data from metagenomics analysis revealed the presence of diverse bacteria, viruses, and fungi. The analyses provide evidence of diverse and highly variable microbial communities in products of animal origin, which is important for food safety, food labeling, biosecurity, and shelf life limiting spoilage by microorganisms.

## 1. Introduction

Changes in food ecosystems and the rise of drug-resistant pathogens [1] has shifted food supply chains to interconnected systems with a variety of complex relationships and exposure to new risks and greater potential of food-borne illness outbreaks [2]. Of the common commodity categories, animal and plant infectious diseases are responsible for major global economic losses in the food and agricultural value chain industries and biodiversity [3]. Early detection of environmental, animal, and plant pathogens is essential in order to prevent and reduce the spread of diseases and facilitate effective management practices. In most low- and middle-income countries, the burden of food-borne diseases has implications on domestic market and international trade [4]. 

While *Listeria monocytogenes*, *Salmonella* spp., Shiga producing toxin *Escherichia coli*, enteric species of *Yersinia* that include *Y. enterocolitica* and *Y. pseudotuberculosis* are listed in World Organization for Animal Health (OIE) as some of the foodborne microorganisms of concern [5], the World Health Organization (WHO) has previously estimated that 31 foodborne diseases (FBDs) resulted in over 600 million illnesses and 420,000 deaths worldwide. Several standards, codes of practice, guidelines, and other recommendations relating to foods, food production, and food safety have been developed under the Codex Alimentarius [6]. Of importance to biological hazards are the general principles of food hygiene; control of *Campylobacter* and *Salmonella* in chicken meat; viruses in food; *Taenia saginata* in meat of domestic cattle; *Trichinella* spp. in meat of Suidae; nontyphoidal *Salmonella* spp. in beef and pork meat, microbiological risk management; minimization and containment of antimicrobial resistance among others [6]. 

The risk for humans to contract foodborne diseases through the consumption of undercooked meat such as rare and blue steaks is a concern. The unexpected recovery of nontuberculous mycobacteria in cooked meat of African buffalo (*Syncerus caffer*) and greater kudu (*Tragelaphus strepsiceros*) suggests possible survival and resistance characteristics of these strains, which is of public health interest [7]. Nearly half of people risk illness from undercooked food, burgers and sausages, and recontaminated ready-to-eat foods [8,9]

Diagnostic microbiology for identification of the microbial contaminants continues to rely upon improved traditional techniques or syndromes of suspected infectious etiology, microscopy, serology, and molecular tools [10]. Cultivation is the most widely used approach in laboratories throughout the world, especially in developing countries [11]. It is well-documented that cultivation of microorganisms does not capture the richness of microbial diversity as many microorganisms thrive in conditions that are not reproducible in laboratory conditions due to varied reasons [10,12,13]. Therefore, some diseases remain poorly understood and inadequately explained from a microbiological perspective, thus, it seems plausible to speculate that the identification of selected pathogens may represent an imperfect understanding of the true diversity of microbes capable of causing human and animal diseases [11,12].

The application of metagenomics for the establishment of comprehensive collection of microbial reference genomes and genes is an important step for accurate characterization of the taxonomic and functional repertoire for the safety and suitability of food systems [14]. Such collection offers a pathway to sustainable healthy food systems through opportunities for predicting the presence of pathogens based on changes observed in entire microbial communities, as well as the potential to characterize unknown microbiota [14]. Broad-range amplification and sequencing of the 16S rRNA gene, directly from field samples, is a method that potentially allows detection of any cultivable or noncultivable bacteria, however, some challenges have been reported as the PCR will amplify all bacterial DNA present, no matter its relevance or not as a pathogen or a contaminant from the sample or the PCR-reagents as the primers are designed to be broad-range [13]. While the bacterial DNA may ‘drown’ in the vast amount of mammalian species DNA which decreases the sensitivity of the assay [10], problems have also been reported with primer cross-reactivity and coamplification of mammalian species mitochondrial DNA, which also contains variants of the 16S rRNA gene [13]. Differences observed in bacterial patterns may therefore be due to extraction methods often caused by the differences in cell lysis efficiency associated with the characteristic cell wall structure of fungi, eukaryotic cells, Gram-positive and Gram-negative bacteria [15].

In South Africa, the Meat Safety Act (MSA), 2000 (Act No. 40 of 2000), provides for measures to promote meat safety and the safety of animal products. The MSA defines “unsafe for human and animal consumption” as unsafe due to a disease, an abnormal condition, putrefaction, decomposition, contamination or residues, or by reason of exposure to or contact with a disease or putrefied, decomposed, or contaminated material. The Red Meat Regulation No. 1072 of 17 September 2004 and the Poultry Regulations No. R. 153 of 24 February 2006 lay down the implementation rules to be complied by food business operators when implementing the general and specific essential national standards and hygiene measures referred to in the Meat Safety Act (Act 40 of 2000). Sections 51 and 52 in the case of poultry regulations and sections 53 and 54 in the case of red meat regulations provide for the owner of an abattoir to divulge a list of all potential biological hazards that may occur, followed by Hygiene Management Programmes (HMP) to prevent, eliminate, or reduce the identified hazards to acceptable levels. Despite these provisions, contamination of food along the value chain is a complicated process, hence regular monitoring and surveillance of microorganisms is paramount [16].

There are some gaps in knowledge regarding the extent of microbial diversity associated with contamination of meat along the food value system in South Africa. This is the first report to present comprehensive findings of diverse foodborne pathogens from meat sourced among different animal species from all the nine provinces of South Africa and imported meat from cold stores at major ports of entry using a large sample size (*n* = 2017 meat and meat products). Therefore, the objectives of this study were: (i) to assess the prevalence of selected bacterial pathogens (*Yersinia enterocolitica*, *Salmonella* species, *Listeria monocytogenes*, *Campylobacter jejuni*, *Campylobacter coli*, *Staphylococcus aureus*, *Clostridium perfringens*, *Bacillus cereus*, and *Clostridium botulinum*) isolated using selective culture methods targeting specific organisms; (ii) to assess the microbial communities of selected subsamples through the application of next generation sequencing. Due to international trade and travel, the findings of this study are important to the international scientific community because contamination of meat and meat products by foodborne pathogens is a global health issue.

## 2. Materials and Methods

### 2.1. Study Area and Design

A cross-sectional study was undertaken to determine the prevalence of *L. monocytogenes*, *Campylobacter* species, *B. cereus*, *C. perfringens*, *Salmonella* species, *Y. enterocolitica*, *S. aureus*, and *C. botulinum* from meat and meat products in all the provinces of South Africa. Samples were collected from October 2014 to December 2016. The samples were from bovine, ovine, caprine, poultry, and game meat. In order to minimize confounding, simple random sampling was used to collect the meat samples from abattoirs, meat-processing plants, butcheries, and retail outlets. The main categories of meat types were raw meat, processed meat products, and ready-to-eat meat.

In order to enhance the robustness of this study, 2017 meat and meat products were analyzed (*n* = 1758 from South Africa; *n* = 259 imported meat samples). Imported meat samples from various countries were collected from Durban, Port Elizabeth, and Western Cape ports of entry cold stores. 

A selection of meat samples from four different categories of processed meat products collected for analysis namely minced meat (*n* = 48), burger patties (*n* = 30), biltong (*n* = 28), and raw sausages (*n* = 35) were sent to the Biotechnology Platform, Onderstepoort, South Africa for amplicon metagenomics analysis intended for mammalian species identification. Some of the samples included information on which species they were produced from and of these 24 were ‘beef mince’, 21 were ‘beef patties’, 19 were ‘beef biltong’, and 25 were ‘beef sausages’. All samples for metagenomic analysis were stored at −20 °C following collection. 

### 2.2. Analysis of Targeted Bacteria by Selective Culture-Dependent Methods

#### 2.2.1. *Listeria monocytogenes*

*L. monocytogenes* were isolated and identified using the *Listeria* Precis method as described by Matle et al. [17].

#### 2.2.2. *Campylobacter* Species

*Campylobacter* species were isolated and identified according to the ISO 10272-1: 2006 protocol. Briefly, selective enrichment for *Campylobacter* species was undertaken by homogenization of the meat and meat products in Bolton broth (1:10 *w*/*v*), followed by incubation in microaerobic conditions for approximately 4−6 h at 37 ± 1 °C. The inoculated Bolton broths were further incubated at 41.5 ± 1 °C for 44 ± 4 h. Loopfuls of inoculated Bolton broths were inoculated onto Modified charcoal, cefoperazone, desoxycholate (mCCD) agar and Butzler agars, followed by incubation in a microaerobic environment (created using Campy gas generating kits) at 41.5 ± 1 °C for 44 ± 4 h. Presumptive *Campylobacter coli* colonies appeared to be glossy, creamy grey moist, and approximately 1.0–2.5 mm in diameter. Presumptive *C. jejuni* were flat, grey-white colonies that were approximately 2.0–3.0 mm in diameter and some were efflorescent. These colonies were subjected to Gram stain and oxidase test for preliminary confirmation. Gram-negative, spiral-shaped rods that were oxidase positive were subjected to confirmation using real-time polymerase chain reaction.

#### 2.2.3. *Bacillus cereus*

*Bacillus cereus* was isolated from meat and meat products according to ISO 7932:2004 protocol, which is a horizontal method for the enumeration of presumptive *B. cereus*–colony-count technique at 30 °C. Meat and meat products were homogenized in maximum recovery diluent (1:10 *w*/*v*), followed by 10-fold dilutions up to 10^−5^. Each sample dilution was surface inoculated onto Mannitol Yolk Polymyxin (MYP) agar in duplicate plates, followed by incubation at 30 ± 1 °C for 18−24 h. In instances where the bacterial colonies were not clearly visible, the inoculated MYP plates were incubated under the same conditions for an additional 24 h. Colonies that were mannitol-negative, hence appeared pink to red and had a zone of precipitate around the colonies (indicating lecithinase positive reaction) were considered presumptive *Bacillus cereus*. The presumptive colonies of *B. cereus* were streaked onto Sheep Blood Agar to evaluate hemolysis. Colonies that exhibited beta-hemolysis were considered presumptive for *B. cereus*. The presumptive *B. cereus* were subjected to a battery of biochemical tests including acid production from phenol red glucose broth, nitrate reduction, acetylmethyl-carbinol production using Voges Proskauer (VP) medium, and production of acid from mannitol. 

#### 2.2.4. *Clostridium perfringens*

*Clostridium perfringens* was isolated from meat and meat products according to ISO 7937:2004, which is a horizontal method for the enumeration of *C. perfringens* using the colony-count technique. Meat and meat products were homogenized in maximum recovery diluent (1:10 *w*/*v*), followed by 10-fold dilutions up to 10^−5^. The diluted samples (1 mL each) were poured into Petri dishes, followed by addition of Tryptose Sulfite Cycloserine (TSC) agar at (44–47 °C) and thorough mixing. Rotation overlay (10 mL Perfringens Agar) was added, followed by incubation at 37 ± 1 °C under anaerobic conditions for 20 ± 2 h. Presumptive *C. perfringens* appeared as black colonies surrounded by opaque white zones approximately 2–4 mm due to lecithinase activity. These presumptive colonies were inoculated into Fluid Thioglycollate Medium, followed by incubation at 37 ± 1 °C in an anaerobic atmosphere for 18∓24 h. For confirmation of identity, the presumptive *Clostridium perfringens* were inoculated in Lactose Sulphite Medium, followed by incubation at 46 °C in a water bath for 18∓24 h. Furthermore, the presumptive *C. perfringens* were inoculated in Nitrate Motility Medium, Lactose Gelatin Medium, followed by incubation at 37 ± 1 °C in an anaerobic atmosphere for 18–24 h. *C. perfringens* were nonmotile, reduced nitrates to nitrites (in Nitrate Motility Medium), and produced gas and acid in Lactose Gelatin Medium. 

#### 2.2.5. *Salmonella* Species

Meat and meat products were analyzed for the presence of *Salmonella* spp. according to ISO 6579, 2002. Briefly, pre-enrichment for *Salmonella* species was undertaken by homogenization of the meat and meat products in buffered peptone water (BPW; 1:10 *w*/*v*), followed by incubation at 37 ± 1 °C for 18 ± 2 h. For selective enrichment, the BPW was inoculated in Rappaport Vassiliadis (RVS) and Müller–Kauffmann tetrathionate (MKTT) broths, followed by incubation for 24 ± 3 h at 41.5 ± 1 °C and 37 ± 1 °C, respectively. Loopfuls of the inoculated RVS and MKTT broths were streaked onto Xylose Lysine Desoxycholate (XLD) and Brilliant Green (BG) agars, followed by incubation at 37 ± 1 °C for 24 ± 3 h. Presumptive *Salmonella* spp. appeared as pink-red black centered colonies on XLD and pink colonies on BG agar. Five colonies of the presumptive *Salmonella* isolates were selected per plate (where available) in order to evaluate whether different serovars were present in one sample. Therefore, for every positive sample, five colonies were selected for further analysis, unless there were fewer colonies present. If less than five colonies were present, they were all subjected to further tests. The presumptive *Salmonella* isolates were confirmed according to ISO 6579, 2002. The confirmed *Salmonella* isolates were purified on Blood Tryptose Agar and incubated at 37 ± 1 °C for 18–24 h, followed by serotyping. *Salmonella* spp. serotyping was done as described in the White–Kauffmann–Le Minor scheme [18,19]. 

#### 2.2.6. Isolation and Identification of *Staphylococcus aureus*

The meat and meat products were analyzed for the presence of *S. aureus* according to ISO 6888-1:1999 + A1:2003. Briefly, 10-fold dilutions of the homogenized meat samples were inoculated onto Baird Parker agar in duplicate, followed by aerobic incubation at 37 ± 1 °C for 18–24 h. Plates with greater than 300 colony forming units for the highest dilution were considered as too numerous to count (TNTC). Typical colonies were selected and tested for the presence of catalase. Furthermore, catalase-positive isolates were evaluated for the presence of coagulase using slide and tube agglutination tests. The coagulase positive isolates were streaked on Mannitol Salt agar to evaluate fermentation of mannitol. In addition, the identity of *S. aureus* was further verified using API-STAPH (bioMerieux, Johannesburg, South Africa).

#### 2.2.7. Isolation and Identification of *Yersinia enterocolitica*

The presence of *Y. enterocolitica* in meat and meat products was evaluated according to ISO 10273:2003, which describes the horizontal method for the detection of *Y. enterocolitica*. Briefly, the samples were diluted in Peptone sorbitol bile broth (PSBB), followed by inoculation on Celfsulodin-irgasan-novobiocin (CIN) and MacCkoneky agar. The inoculated plates were incubated aerobically at approximately 30 °C for 24–48 h. In addition, the homogenates were incubated at 10 °C for 10 days, followed by streaking on CIN and MaCkonkey agar plates and incubation at 30 ± 1 °C for 24–48 h. Colonies that appeared small with deep red centers and clear colorless zones surrounding the colonies on CIN and small colorless on MacConkey agar were considered presumptive and they were subjected to biochemical tests. The biochemical tests involved testing for urease production on Christensen’s Urea agar, and aesculin production on Bile Esculin agar. The other biochemical tests were indole test, Methyl Red-Voges Proskauer test, citrate test and fermentation of mannitol, sorbitol, rhamnose, raffinose, trehalose, salicin, and xylose.

#### 2.2.8. Isolation of *Clostridium botulinum*

Analyses of samples for *C. botulinum* was undertaken using strict anaerobic conditions. The samples for analyses of *C. botulinum* were specifically placed at the bottom of cooked meat medium (Oxoid) immediately after collection. The inoculated samples were incubated at 35 ± 1 °C for 5 days under anaerobic conditions. The samples were evaluated for gas production, turbidity, and for possible digestion of the meat particles. Loopfuls of the broths were Gram-stained and evaluated for the presence of cells that appeared ‘racket shaped’ due to clostridial cells. Loopfuls of broth cultures were steaked onto Blood Tryptose agar (BTA; Onderstepoort Biological Products, Pretoria, South Africa), followed by incubation at 35 ± 1 °C in anaerobic atmosphere created using anaerobic gas generating kits (Oxoid, Basingstoke, UK) for 48–72 h.

#### 2.2.9. Reference Strains

Both positive and negative reference cultures were included alongside meat samples for all experiments to ensure quality control and validity of the results. The following reference strains were used as positive controls in this study: *S*. Typhimurium ATCC 14028, *Staphylococcus aureus* ATCC^®^ 25923™, *C. coli* ATCC 33559, *C. jejuni* ATCC 33560, *C. lari* ATCC 35211, *B. cereus* ATCC 14579, and *Clostridium perfringens* ATCC^®^ 13124™. *L. monocytogenes* ATCC19111 was used as positive control whilst *Escherichia coli* ATCC 25922 was used as negative control as described by Matle et al. [17].

### 2.3. Identification of Isolates Using Molecular Techniques

#### DNA Extraction and Real-Time PCR

The foodborne pathogens that were confirmed using biochemical tests were revived in appropriate broth media, followed by checking for purity on Blood Tryptose agar and Gram stain. Furthermore, DNA was extracted using QIAGEN DNeasy Blood and Tissue kit (QIAGEN, Damstradt, Germany) according to the manufacturer’s instructions. The DNA that was extracted using QIAGEN DNeasy Blood and Tissue kit (QIAGEN, Damstradt, Germany) was quantified by using the NanoDrop Instrument (NanoDrop Technologies, Wilmington, DE, USA) and the quality was confirmed using agarose gel electrophoresis (0.8% agarose).

Real-time PCR was used for two purposes, namely: (i) to confirm the identity of *L. monocytogenes, Salmonella* spp., and *C. jejuni*, *C. coli*, and *C. lari* pure cultures; (ii) to detect *L. monocytogenes, Salmonella* spp., and *C. jejuni*, *C. coli*, and *C. lari* directly from the meat samples without culture. The protocol for the two approaches was similar with the exception of the method used for DNA extraction. The DNA from pure cultures was extracted using QIAGEN DNeasy Blood and Tissue kit (QIAGEN, Damstradt, Germany), whereas direct extraction of bacterial DNA from the meat samples was extracted using the appropriate PrepSEQ^®^ NucleicAcid Extraction Kit according to the manufacturer’s instructions. Each PCR tube contained an internal positive control (contained in the lyophilized pellet). RT-PCR results for *L. monocytogenes*, *Salmonella* spp. were interpreted using RapidFinder™ Express Software, which was installed in the 7500 Fast Real-Time PCR System (Applied Biosystems, Foster City, CA, USA).

*L. monocytogenes, Salmonella* spp., and *C. jejuni*, *C. coli*, and *C. lari* were detected using Applied Biosystems^®^ 7500 Fast Real-Time PCR System (Applied Bio-systems, Foster City, CA, USA) according to the manufacturer’s instructions. The *L. monocytogenes* was detected using MicroSEQ *Listeria monocytogenes* pathogen detection kit (Applied Biosystems, Foster City, CA, USA) according to the manufacturer’s instructions. *Salmonella* spp. was detected using the MicroSEQ^®^ *Salmonella* spp. detection kit according to the manufacturer’s instructions. Real-Time PCR for simultaneous detection of *C. jejuni*, *C. coli*, and *C. lari* from food samples was verified using the RapidFinder™ *Campylobacter* Multiplex Assay Beads. The *Campylobacter* Multiplex Assay is not AFNOR-validated and Sequence Detection System (SDS) software on the 7500 Fast Real-Time PCR System was used to interpret results.

### 2.4. Identification of Microbial Genomes from Collected Product Samples (Metagenomics Analyses)

#### 2.4.1. DNA Extraction

Genomic DNA for the tests was extracted from 300 mg of each processed meat sample submitted to the laboratory using a Hamilton Star Plus automated liquid handler (Hamilton Inc.) to cater for the sampling size. Genomic DNA from the meat samples used for verification test was extracted manually from 40 mg of pure meat samples. A Macherey—Nagel kit (Macherey—Nagel, Germany) was used for DNA extraction according to the manufacturer’s protocol. Quantification of DNA for all samples was done using Qubit^®^ fluorescent dye method and gel electrophoresis was used to assess quantity and quality of starting material. 

#### 2.4.2. Quality Control of DNA Extracts

In order to test the specificity of the 16S universal primers and also confirm the origin of the known species, DNA from the nine different species of known origin was amplified and sequenced individually. 

#### 2.4.3. Polymerase Chain Reaction

Polymerase chain reaction (PCR) for the mitochondrial 16S rRNA gene was performed using universal mammalian primers [20] tailed with Nextera adapters (Table 1). Thermal cycling was performed in a Labnet MultigeneTM Gradient Thermal Cycler (Woodridge, IL, USA) at a final volume of 25 µL containing 12.5 µL of 2× Hot start PCR mastermix, 2.5 µL of each forward and reverse primer (1 mM final concentration), 5 µL RNase-free water, and 2.5 µL of DNA template. The PCR conditions were as follows: denaturation at 95 °C for 3 min, followed by 30 cycles of 90 °C for 20 s, 65 °C for 30 s, 72 °C for 30 s, and finalization at 72 °C for 5 min. The PCR products for the mitochondrial 16S rRNA gene were 186 bp in length. The PCR products were subjected to electrophoresis in 2% agarose gels in 1 × tris-acetate-EDTA (TAE) buffer at 90 V for 45 min. The amplified products were visualized under ultra-voilet light in a transilluminator. Purification of PCR products was performed using a Qiagen MinElute^®^ PCR purification kit (Qiagen, Germany) according to the manufacturer’s protocol. Quantification of the purified samples was done using Qubit^®^ fluorescent dye method. The purified products were stored at 4 °C prior to sequencing.

#### 2.4.4. Library Preparation and MiSeq Sequencing

Library preparation was performed using the 16S Metagenomics Sequencing Library Preparation kit according to the manufacturer’s protocol (Illumina, Inc, San Diego, CA, USA). Quality control of the sample library and quantification of the DNA library templates was performed. Quantification of DNA was done using Qubit^®^ fluorescent dye method. The library size distribution was checked using a High Sensitivity DNA chip. Thereafter, the indexed libraries were normalized, pooled, and loaded onto an Illumina MiSeq reagent cartridge using MiSeq reagent kit v3 and 600 cycles. The paired end 2 × 300 bp sequencing was run on an Illumina MiSeq 2000 sequencer at 0.2× coverage at the Biotechnology Platform, Agricultural Research Council, Onderstepoort, South Africa.

#### 2.4.5. Bioinformatics

Quality control, adapter removal, decontamination, and error correction of the raw sequence data was done using BBDuk (version 37.90; https://jgi.doe.gov/data-and-tools/bbtools/bb-tools-user-guide/bbduk-guide/). Taxonomic assignment of the filtered reads was done with two widely used applications. Kaiju [21] is used for the taxonomic classification of high-throughput sequencing reads. Kraken 2 [22] is the latest version of Kraken, and is a taxonomic classification system that uses exact k-mer matches. Results obtained from these two pipelines were visualized using R v.3.6.0 [23] and the ggplot2 package [24].

In the second scenario, the entire database of mitochondrial sequences for 289 different species was also downloaded from GenBank (http://www.ncbi.nlm.nih.gov/nuccore). The DNA sequences from the 16S rRNA genes were extracted using Feature Extract 1.2 Server (http://www.cbs.dtu.dk/services/FeatureExtract). The sequences were in Text format and were converted into FASTA format in CLC Genomics Workbench v.8 and exported into MEGA v.6.06 for phylogenetic analysis. Multiple sequence alignment of these was performed with ClustalW within MEGA v.6.06 using the default settings. In order to visualize the ability of the 16S rRNA gene to separate different species, a neighbor-joining (NJ) tree was constructed using the Kimura 2-parameter model in MEGA v.6.06. The number of bootstrap replications was 1000. The bootstrap analyses show how well supported a tree is, taking into consideration the data inputed and also the method used to construct the tree. The horizontal length of branches indicates the evolutionary distance between organisms. This reveals the number of nucleotide substitutions per site along the branch from the node to the endpoint [25].

In another scenario, the quality of the sequences was checked using FastQC (http://www.bioinformatics.babraham.ac.uk/projects/fastqc/) and trimming for quality control was performed using Trimmomatic (http://www.usadellab.org/cms/?page=trimmomatic), using a Phred score of 33. Therefore, reads with a Phred score of 33 and above were kept. Following trimming FASTQ files were converted to FASTA files and sequence similarity searches were conducted using local BLAST (megablast) against a nucleotide (nt) database downloaded from the NCBI (www.ncbi.nim.nig.gov/nuccore). Following a BLAST analysis, reads that had an alignment length of 100 bp and above and a similarity score of 99% and above were kept. These reads were then imported to Excel to check the percentage of species present in each sample.

## 3. Results

### 3.1. Contamination of Meat and Meat Products Based on Culture Methods and PCR

The results show different bacterial phyla were present in the collected meat and animal product samples. The analysis of bacterial pathogens obtained by the selective culture dependent approach showed isolation and detection of *Y. enterocolitica*, *Salmonella* spp., *L. monocytogenes*, *Campylobacter* species including *C. jejuni*, *C. coli*, and *C. lari*; *S. aureus*, *C. perfringens*, and *B. cereus* (Tables S2–S6). *Clostridium botulinum* was not isolated in any of the samples. The occurrence of *L. monocytogenes* in various meat products in South Africa is described by Matle et al. [17]. Analyses of the bacterial contamination of the meat and meat products by meat type clearly showed that the main source of contamination by foodborne pathogens occurred in raw processed meat for all the detected food borne pathogens (Tables S2–S6). However, no general trend could be deduced for other food establishments. 

Table S2 shows the proportions of *Campylobacter* spp. in meat and meat products from diverse animal species, different establishments and types from all nine provinces of South Africa. Overall, a total of 159 chilled and frozen samples out of 1758 (9.04%) collected meat samples on the domestic market were RT-PCR positive for *Campylobacter* spp. (Table S2). Eight of the 259 frozen samples from ports of entry were positive for *Campylobacter* spp. (Table S2). Raw processed meat showed the highest proportion of *Campylobacter* positive samples (14.38% of raw processed meat; Table S2), whilst ready-to-eat meat and meat product showed the least number that tested positive for *Campylobacter* (2.29% of the ready-to-eat samples; Table S2) and this trend was observed from the nine provinces of SA (Table S2). In general, the majority of *Campylobacter* positive samples were recovered from processing plants (33.33% of samples obtained in processing plants with a range of 0–50% across the nine provinces; Table S2). The proportion of *Campylobacter* contamination based on animal species ranged from 0% (in lamb) to 24.4% in meat from diverse animal species (mixed samples). Poultry meat had the second highest proportion of *Campylobacter* contamination (12%; *n* = 48/400; Table S2). 

Table S3 shows the proportions of *B. cereus* in meat and meat products from different enterprises and meat types from all nine provinces of South Africa. On average, *B. cereus* were isolated from 4.5% (79/1758) of the domestic meat samples, and 2.7% (7/259) of imported meat samples, which yielded 231 isolates (Table S3). Raw processed meat showed the highest proportion of *B. cereus* positive samples (17.19%; 55/765 of raw processed meat; Table S3). This trend was observed from seven of the nine provinces of SA (Table S3). Pork meat and meat products showed highest proportion of *B. cereus* contamination (12.6%; 17/13; Table S3).

Table S4 shows the proportions of *Clostridium perfringens* in meat and meat products from diverse animal species, different establishments, and meat types from all nine provinces of South Africa. *C. perfringens* was isolated from 360 out of 1758 (20.48%) contaminated samples from South African meat and 50 out of 259 (19.31%) *C. perfringens* positive samples were from imported meat (Table S4), and most of these bacteria belonged to toxin type A. Raw processed meat showed the highest proportion of *C. perfringens* positive samples (23.53%; 180/765 raw processed meat; Table S4) and this trend was observed from seven of the nine provinces of SA (Table S4). The proportion of raw-intact meat that tested positive for *C. perfringens* was similar to that of raw processed meat (23.52%; 131/557 of raw processed meat; Table S4). 

Table S5 shows the proportions of *Salmonella* spp. in meat and meat products from different enterprises and meat types from all nine provinces of South Africa. On average, 50 of the 1758 (2.84%) South African meat and meat products, and 13 out of the 259 (5.02%) imported meat were contaminated with *Salmonella* spp. based on isolation techniques and confirmation by biochemical tests, serotyping, and PCR (Table S5). The 63 *Salmonella* positive samples yielded 125 isolates with diverse serotypes including *S.* Typhimurium, *S.* Aarhus, *S.* Anatum, *S.* Heidelberg, *S.* Infantis, *S.* Muenchen, *S.* Daula, *Salmonella* II, *S.* Ohio, *S.* Kingston, *S.* Othmarschen, *S.* Kentucky, *S.* Muenster, *S.* Glostrup, *S.* Sandiego, *S*. Derby, *S*. Ivory, *S.* Bovismorbificans, *S.* Yaba, *S.* Jerusalem, *S.* Schwarzengrund, *S.* Tees, *S.* Hull, *S.* Soahanina, *S.* Eastbourne, *S.* Haifa, *S.* Kentucky, *S.* Mampeza, *S.* Stanleyville, *S.* Wangata that were isolated from the South African market. *S.* Enteritidis, *S.* Heidelberg, *S.* Aarhus, *S.* Kentucky, and *S.* Wippra were isolated from chicken meat at the ports of entry. Raw processed meat showed the highest proportion of *Salmonella* positive samples (3.9%; *n* = 30/765 of raw processed meat; Table S5).

Table S6 shows the proportions of *Yersinia enterocolitica* in meat and meat products from diverse animal species, different establishments and meat types from all nine provinces of South Africa. On average, *Y. enterocolitica* was isolated from 410 of the 2017 samples (20.35%), of which 360 of the positive samples were from South Africa (17.87%; Table S6). Raw processed meat showed the highest proportion of *Y. enterocolitica* positive samples (30.07%; 230/765 of raw processed meat; Table S6), whilst ready-to-eat meat and meat products showed the least number that tested positive for *Y. enterocolitica* (2.83%; 13/436 of the ready-to-eat samples; Table S6) and this trend was observed from all the nine provinces of SA (Table S6). 

*S. aureus* showed contamination rate of approximately 62.57% (1100/1758) from meat and meat products placed on the domestic market in South Africa and 38.61% (100/259) for imported meat at the ports of entry (Table S7). The highest contamination was observed from raw processed meat at 72.55% (555/765) Table S7), whereas 33.26% (145/436) of the products that tested positive for *S. aureus* were ready-to-eat meat products (Table S7). The majority of positive samples on the domestic market showed counts that were less than 2 Log CFU/g (35%; *n* = 385/1100), followed by 3 Log CFU/g (34, 09%; *n* = 375/1100) and 62 (5, 64%) of the samples revealed counts from greater than 3 Log CFU/g to 5 Log CFU/g. Even so, 26.18% (*n* = 288/1100) of the *S. aureus* positive meat and meat products had counts that were considered too numerous to count (TNTC), with raw processed meat constituting the majority of these samples (20%; *n* = 220/1100) and RTE meat products showing the lowest proportion of positive samples (0, 73%; *n* = 8/1100). The *S. aureus* counts for imported meat samples showed an almost similar trend with the majority of samples consisting of counts that were less than 2 Log_10_ (40%; *n* = 40/100), followed by 3 Log_10_ CFU/g (30%; *n* = 30/100). The proportions of imported samples with *S. aureus* cells with counts from greater than 3 Log_10_ CFU/g to 5 Log_10_ constituted 15% of the 100 positive samples and 15% (*n* = 15) of the samples had counts that were considered too numerous to count.

### 3.2. Contamination of Meat and Meat Products as Revealed by Metagenomics Analyses

Results on the several animal species identified from the samples collected and analyzed are not presented as they are a focus of another publication. Although bias has been reported with the use of metagenomic analyses, the available tests are able to capture microbial diversity by directly analyzing the sample genetic material without the need for culturing [26]. The data was screened for the presence of DNA signatures of potential agents using various software platforms to perform the analyses so that the results did not depend on a single taxonomic profiling tool. The DNA signature hits observed highlights the functionality and power of sequencing-based approaches to identify microorganisms within the value chain without the need for culturing. 

**Figure 1:**
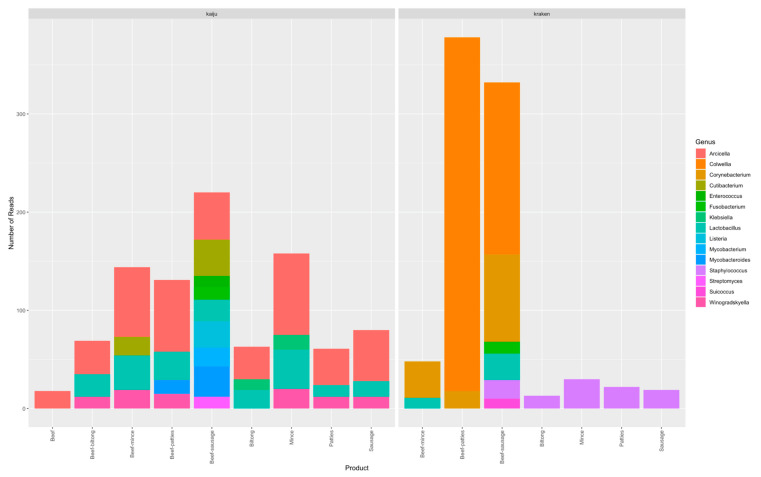
Number of reads (*n* ≥ 10) assigned to different genera obtained from various product types.

The Kaiju protocol assigned reads of the 15 product types to 93 different genera. The highest read count (83) was assigned to the genus *Arcicella* and found in mince (Supplementary Table S1). Kraken 2 assigned reads to 114 different genera in 13 product types with the highest read count (360) belonging to the genus *Colwellia* obtained from beef-patties (Supplementary Table S1). The samples that yielded the top three number of reads above 10 were the sausages, mince, and patties. The results are displayed in Figure 1.

The BLAST findings also suggest the presence of DNA signatures of potential pathogenic species, including *Staphylococcus aureus*, *Legionella waltersii*, *Clostridium botulinum*, *Clostridium tetani*, *Streptococcus agalactiae*, *Bacillus infantis*, African Swine Fever Virus, *Aerococcus urinae*, *Chryseobacterium shandongense*, *Orientia tsutsugamushi*, *Micrococcus luteus*, *Burkholderia contaminans*, *Delftia acidovorans*, *Corynebacterium atypicum*, *Corynebacterium camporealensis*, *Corynebacterium endometrii*, *Corynebacterium diphtheriae*, *Staphylococcus haemolyticus*, *Staphylococcus pseudintermedius*, *Streptococcus pluranimalium*, *Avibacterium volantium*, *Campylobacter hyointestinalis*, *Arcanobacterium haemolyticum*, *Campylobacter concisus*, *Campylobacter sputorum*, *Moraxella bovoculi*, *Dichelobacter nodosus*, *Neisseria animaloris*, *Salmonella enterica*, *Escherichia coli*, *Streptococcus pluranimalium*, *Brachybacterium paraconglomeratum*, *Fusarium chlamydosporum*, and *Curvularia aeria*. Several of the detected microbial organisms have not been cultured in the laboratory and their significance in food remains unknown, highlighting that little information is known about the safety and suitability of food products in the food ecosystem and One Health triad. 

## 4. Discussion

The 2015 WHO report highlighted 31 most frequent causes of foodborne diseases including bacteria, viruses, parasites, toxins, and chemicals. This study provides insight on the findings of meat contamination by selected foodborne pathogens based on culture dependent approaches, which represent a relatively small proportion of the total food microbial diversity and the importance of using culture independent studies, which allows identification of uncultured and novel taxa within the food microbiota [27,28]. The selective culture dependent methods resulted in the isolation of targeted microorganisms, which were less in terms of diversity compared to what was revealed by the metagenomics analysis. 

In this study, culture-based methods revealed that meat and meat products from different animal species were contaminated with at least one of the tested foodborne pathogens (*Yersinia enterocolitica*, *Salmonella* species, *Listeria monocytogenes*, *Campylobacter jejuni, Campylobacter coli*, *Staphylococcus aureus, Clostridium perfringens*, *Bacillus cereus*) except *C. botulinum*. This is concerning from a public health standpoint because some of the foodborne pathogens have been linked to serious foodborne outbreaks and fatalities [29]. Furthermore, the isolation of these foodborne pathogens from meat and meat products in South Africa highlights the importance of implementing a One Health multifaceted, transdisciplinary, and collaborative cross-sectorial strategy involving different activities such as ensuring healthy animals, a healthy ecosystem, research, surveillance at farm and production levels, data sharing, and standardization of testing protocols across different sectors in order to minimize risk of foodborne infections and enhance public health outcomes [30,31,32]. 

The proportion of samples that tested positive in raw processed meat from this study was relatively high compared to raw intact and ready-to-eat food. This is probably due to further contamination that occurs as a result of contact with contaminated surfaces, contaminated hands of meat handlers, or even contaminated clothing. Vigilance is therefore required at every stage of meat processing and regular surveillance in order to establish whether established HACCP and GHP are in harmony with empirical evidence provided by microbiological findings. There were some differences that were observed between the extent of contamination of locally produced meat samples from South Africa and imported samples at the cold stores at major ports of entry. However, caution should be exercised when interpreting these findings because samples collected on the domestic market were likely to have been relabeled and be in combination with and/or in contact with imported products of plant and animal origin. Even so, the information about microbial contamination from outside South Africa provides insights about possible sources of microbial populations on the local food market and their potential contribution to possible infections.

*L. monocytogenes* has been reported in several countries, and its incidence depends on eating habits, cooking practices, use of refrigeration, and food importation. The significant role of *L. monocytogenes* as a foodborne pathogen is evident from the valuation costs of fatality rates in human population. Based on publicly available information at the time during the outbreak, the partial cost of the listeriosis outbreak observed in South Africa (*L. monocytogenes* sequence type 6 (ST-6)) in 2017–2018 due to polony was estimated at a minimum of USD 260 million [33]. When other livestock value chains are considered, on a worst case scenario, the modeled overall negative economic impact during the 2017/2018 human listeriosis outbreak in South Africa was estimated to be USD 2.3 billion (R39.8 billion) amounting to 0.82% of the Gross Domestic Product (GDP) with government response costs at R65.5 million, however, the overall impact figures are likely to be lower considering a growing body of better data quality [29]. Occurrence, serotypes, and characteristics of *L. monocytogenes* in meat and meat products in South Africa and implications for the food industry and public health has been well-described [17]. 

A total of 159 chilled and frozen samples out of 1758 (9.04%) collected meat samples on the domestic market were culture and RT-PCR positive for *Campylobacter* spp. Eight of the 259 frozen samples from ports of entry were positive for *Campylobacter* spp. In general, studies on *Campylobacter* species focused on a single animal species, and sample size calculations were not stipulated per species level, hence direct comparison with our study has some limitations. Even so, it is important to make a comparison of this study with related studies in order to obtain context. The average proportion of *Campylobacter* contamination of meat and meat products from this study was generally lower than the prevalence of *Campylobacter* that was observed in retailers from Kenya, where Carron and coworkers [34] observed contamination between 60% and 64% of poultry in retailers and prevalence between 33% and 44% among broiler and indigenous chicken farms, respectively. The proportion of *Campylobacter* in poultry from this study (12%; 48/400 poultry samples) was relatively higher compared to the occurrence of poultry meat preparations at retail shops and processing plants in Italy (5.7%; 12/209) [35]. This could be probably explained by the differences in sample size and/or differences in processing along the meat value chain. The proportion of beef contaminated with *Campylobacter* species in this study was 8.17% (88/1077 of beef samples) compared to a prevalence of 14.2% of the 120 samples of raw beef from wet market and 7.5% of the 120 samples of raw beef from the hypermarket in Selangor, Malaysia [36]. Although there are differences in sample sizes, our findings are almost similar to prevalence of *Campylobacter* contamination from the study that was undertaken on the hypermarket in Selangor, Malaysia [36]. 

*Campylobacter* is one of the leading causes of diarrheal disease for individuals who travel to developed countries [37]. The predominance of *Campylobacter* spp. in poultry in this study necessitates the application of guidelines on *Campylobacter* and *Salmonella* as specified in the Codex Alimentarius [6]. In the European Union, campylobacteriosis was the most commonly reported zoonosis in the EU with an upward trend since 2008, but stabilized during 2013–2017 [38]. In Limpopo province of South Africa, *C. jejuni* was detected in 10.2% and 20.3% of stool samples collected from patients admitted to a hospital and people in rural areas in the northern most district of Vhembe [39]. Campylobacteriosis is known to cause gastroenteritis and *C. jejuni* may progress to other serious conditions [40]. Further, in two interrelated studies undertaken in a Durban hospital, *Campylobacter* was found in 21% of the stool samples taken from 126 malnourished inpatient children compared with 7% of the stool samples taken from 352 randomly selected outpatient children [41]. The infective dose of *C. jejuni* is considered to be low and acute illness from *C. jejuni* may require high doses while infection occurs at low doses [42]. In outbreaks, illness occurs at low doses, while in challenge studies high doses may be required [42]. Human feeding studies suggest that about 500–800 bacteria may cause illness in some individuals, while in others, greater numbers are required [42]. However, it has been speculated further that the dose of *C. jejuni* required for the development of campylobacteriosis can be as low as 360 CFU/g [35,43,44]. Mathematical modeling suggested that an intermediate dose of 9 × 10^4^ CFU/mL has the highest ratio of illness to infection or is considered the optimum infective dose [45]. 

*Y. enterocolitica* are widely distributed in the environment and some aetiological agents of human illness have been isolated from poultry and pigs [46]. Compared to other common foodborne pathogens, the infective dose of pathogenic *Y. enterocolitica* is higher and is estimated at 10^8^–10^9^ cells [37]. In this study *Y. enterocolitica* was isolated from 410 of the 2017 samples (20.35%), of which 360 of the positive samples were from South Africa (17.87%). The possible challenge posed by *Y. enterocolitica* is that the bacteria may grow and survive even in foods that are stored in the refrigerator [47]. A study that was undertaken in Egypt on the prevalence and characteristics of *Y. enterocolitica* from retail and processed meats yielded comparable results with our study to a certain extent [48]. A relatively small sample size of 210 samples that were collected in Mansoura city, Egypt revealed *Y. enterocolitica* prevalence of 14.29%, which was contributed by 15.83% from chicken meat, 10% from ground beef, 16.67% from beef burger, and 10% from sausage samples [48]. A study on the prevalence and characteristics of *Y. enterocolitica* in retail poultry meat (*n* = 500) and swine feces (*n* = 145) in some areas of China showed a much lower *Y. enterocolitica* prevalence of 4.8% in retail poultry meat and 2.76% in swine feces [49]. The differences could be due to differences in storage and handling conditions. However, a previous study in 24 provincial capitals of China (July 2011 to May 2014) that was aimed at systematic evaluation of the prevalence and characteristics of *Y. enterocolitica* in 455 diverse frozen food samples (chicken-meat, duck-meat, pork, beef, sheep-meat, ham, and frozen pasta) revealed 12.3% (*n* = 56) contamination, ranging from 8.9% in frozen pasta samples to 24.2% in frozen sheep meat [50]. The results illustrate a need to expand the scope of food surveillance.

*C. perfringens* was isolated from 360 out of 1758 (20.48%) contaminated samples from South African meat and 50 out of 259 (19.31%) *C. perfringens* positive samples were from imported meat and most of the bacteria belonged to toxin type A. A study that was undertaken between June and September 2015 on the prevalence and toxin types associated with *C. perfringens* recovered from beef from diverse meat markets in Seoul, Korea showed lower prevalence of 4.88% (*n* = 4; based on culture) and 12.20% (*n* = 10; based on RT-PCR) compared to the average proportion of *C. perfringens* from this study [51]. However, the sample size was much smaller (*n* = 82), compared to the 2017 samples that were analyzed in this study. The proportion of raw processed and raw intact meat and meat products on the domestic market that tested positive for *C. perfringens* in this study was approximately 24%, which was almost double the prevalence of positive samples that was observed in Seoul, Korea. Even so, it is important to exercise caution when making comparisons due to the differences in sample sizes, animal species, and techniques that were used. Despite the differences in the proportions of samples that tested positive, it is important to remain vigilant during processing of meat products because *C. perfringens* is associated with diverse environments including soils, food, sewage and contains spores that are challenging to destroy [52,53]. As a member of the gastrointestinal (GI) tract microbiota and a fast-growing pathogen, *C. perfringens* has been reported to secrete greater than 20 virulent toxins [52,53,54]. There is no clarity on the infective dose of *C. perfringens*, but if large numbers of vegetative bacteria or spores are ingested, signs of illness occur [55].

*B. cereus* were isolated from 4.5% (79/1758) of the domestic meat samples, and 2.7% (7/259) of imported meat samples, which yielded 231 isolates. The proportion of samples that tested positive for *B. cereus* in this study is generally low, which is in contrast to the findings of a study on the prevalence and characteristics of *B. cereus* among ready-to-eat foods from retail markets and supermarkets in China that reported 35% (*n* = 860) prevalence among ready-to-eat foods [56]. In this study, 2.75% (12/436) of the ready-to-eat meat products tested positive for *B. cereus.* Even so, the contaminated samples may pose risk to consumers because there is no further processing prior to consumption of these foods. Furthermore, *Bacillus cereus* is a facultative anaerobic Gram-positive bacterium that forms spores andproduces toxins [57]. When present in foods there are intestinal or nonintentional illnesses associated with the production of tissue-destructive exoenzymes [58]. The infectious dose for both the diarrheal and emetic syndromes are >10^5^ cells [59].

*S. aureus* showed the highest average contamination rate of approximately 1100 of the 1758 (62.57%) South African samples and 100 of the 259 (38.61%) imported samples. High proportions of *S. aureus* contamination have been observed in other studies. For instance, *S. aureus* tested positive among 68% of 50 samples in a study in marketed red meat in Nepal [60]. However, direct comparison with the current study should be done with caution because of the large differences in sample size. The average proportion of *S. aureus* positive meat and meat products from this study were higher compared to 35.0% (647/1850) *S. aureus*-positive retail meat and meat products that was observed in China in a study that was undertaken from July 2011 to June 2016 [61]. However, the proportion of *S. aureus* positive samples was varied for different meat types. For instance, Wu and coworkers [61] observed that 60.9% of the quick-frozen poultry tested positive for *S. aureus*, which is above the proportion observed in this study. A study on evaluation of the prevalence and characteristic of *S. aureus* from raw and grilled beef in Ghana yielded lower *S. aureus* combined prevalence of 16.67% (9/54) compared to findings from this study [62]. However, the sample size of 54 that was used by Adzitey and coworkers [62] was much smaller compared to the 2017 samples that were used in this study, hence a direct comparison may be challenging. Despite the variations in findings, the presence of *S. aureus* in raw, processed, and ready-to-eat foods may indicate some inadequacies in hygiene, inappropriate food handling, contamination after processing, or contaminated environment [60]. Staphylococcal food poisoning is an intoxication that is caused by the ingestion of food containing preformed enterotoxin [63]. *S. aureus* produces diverse toxins and invasive enzymes, hemolysins, Panton-Valentine leukocidin, toxic shock syndrome toxin-1, plasma coagulase, as well as deoxyribonuclease [64]. Inappropriate temperature enables the growth and production of enterotoxin at concentrations that are adequate to produce symptoms. The minimum dosage of SE that causes an illness is approximately 10^5^–10^8^ CFU/g of *S. aureus* [65]. The safety margin in risk management for *S. aureus* is dependent on the values of lag time and specific growth rate, which are influenced by temperature, pH, and sodium chloride among other factors. In this study, 26.18% (*n* = 288/1100) of the meat and meat products had *S. aureus* counts that could pose a risk of SE production. The high *S. aureus* counts could be due to breakdown in hygiene at some point of the meat value chain or inappropriate storage conditions [66,67]. Vigilance is paramount during processing meat because raw processed meat constituted the majority (20%; *n* = 220/1100) of potentially risky category of meat. Although RTE meat and meat products showed the lowest proportion of positive samples, *S. aureus* positive samples (0.73%; *n* = 8/1100) pose a challenge and may be a high risk to consumers because there is no further treatment. 

Human salmonellosis is one of the most common and economically important zoonotic diseases [68]. The ability to adapt to the conditions in the host organism and the resultant pathogenicity depend on the serotype with *S.* Typhi and *S.* Paratypahi being pathogenic for humans and asymptomatic in animals while *S.* Cholerasuis is carried mostly by pigs but may cause salmonellosis in humans [69]. There have been previous reports on *Salmonella* serotypes and antimicrobial resistance profiles in the animal protein value chain in South Africa [16]. The importance of *S.* Typhimurium, and *S.* Enteritidis is known, nevertheless, there is a clear record of foodborne outbreaks in South Africa associated with other *Salmonella* serovars [70]. In this study, on average, 50 of the 1758 (2.84%) South African meat and meat products, and 13 out of the 259 (5.02%) imported meat were contaminated with *Salmonella* spp. based on isolation techniques and confirmation by biochemical tests, serotyping, and PCR. These 63 *Salmonella* positive samples yielded 125 isolates with diverse serotypes including *S.* Typhimurium, *S.* Aarhus, *S.* Anatum, *S.* Heidelberg, *S*. Infantis, *S*. Muenchen, *S*. Daula, *Salmonella* II, *S.* Ohio, *S.* Kingston, *S*. Othmarschen, *S*. Kentucky, *S*. Muenster, *S*. Glostrup, *S*. Sandiego, *S*. Derby, *S*. vory, *S*. Bovismorbificans, *S.* Yaba, *S*. Jerusalem, *S*. Schwarzengrund, *S*. Tees, *S*. Hull, *S*. Soahanina, *S*. Eastbourne, *S*. Haifa, *S*. Kentucky, *S*. Mampeza, *S*. Stanleyville, *S*. Wangata that were isolated from the South African market. *S*. Enteritidis, *S.* Heidelberg *S*. Aarhus, S. Kentucky, and *S.* Wippra were isolated from imported chicken meat. Outbreak investigations show the infective dose ranges between 10^6^ and 10^8^ cells, but in some people, even the dose of 10 cells may lead to the development of salmonellosis [71]. 

Different studies in diverse geographical areas revealed diverse *Salmonella* prevalence in meat and meat products, which were generally high compared to the current study. Studies in different African countries showed different prevalence in meat from different animal species [16,72,73]. A study in Nepal that involved analyses of diverse foodborne pathogens yielded a high proportion of *Salmonella* in meat (34% of 50 samples) [60]. The variations in prevalence in *Salmonella* in meat from different geographical areas could be due to differences in study design, isolation methods, and hygiene practices along the meat value chain in the different settings. In a number of studies, serotyping, which is important from an epidemiological standpoint was not done, hence comparison of the potential risk of the *Salmonella* isolates from these studies is challenging. *Salmonella* contamination along the meat production chain from the farm to the consumer can be caused by transportation, inadequate sterilization of utensils, equipment, and contaminated hands of personnel [16]. This necessitates vigilance to be practiced along the entire meat value chain in order to curb the risk of human salmonellosis. 

### 4.1. Contamination Observed from Metagenomics Using Data Kaiju and Kraken Protocols

Some of the microbiota detected in the current study are listed in South Africa as microorganisms of potential concern in the Hazardous Biological Agents regulations under the Occupational Health and Safety Act, 1993 (Act No 85 of 1993). Notifiable medical conditions are stipulated in Regulation No 1434 under the National Health Act, 2003 (Act No 61 of 2003) and the Declaration of certain biological goods (human pathogens, plant pathogens, selected genetically modified organisms, animal pathogens, zoonosis, and toxins) is stipulated in regulation 494 under the Non Proliferation of Weapons of Mass Destruction Act, 1993 (Act No 87 of 1993). This study brings an understanding of the natural ecology of microbes in foods as the growth, survival, and activity of one species is determined by the presence and interactions of other species. It is also clear from the study that previously, only a small percentage of bacterial species in food had been discovered and reported. The identification of sequences assigned to diverse microbiota such as *Staphylococcus*, *Legionella*, *Clostridium*, *Streptococcus*, *Bacillus*, *Aerococcus*, *Chryseobacterium*, *Orientia*, *Micrococcus*, *Burkholderia*, *Delftia*, *Corynebacterium*, *Avibacterium volantium*, *Campylobacter*, *Moraxella*, *Dichelobacter*, and *Neisseria* among others is of significance as the individual bacterium or in combination have been previously associated with either adverse plant health, animal health, food spoilage, and/or food safety elsewhere [74]. *L. waltersii* has been identified as a cause of severe human pneumonia but is not detected by routine laboratory tests. *Aerococcus urinae* is an emerging cause of urinary tract infection in older adults with multimorbidity and urologic cancer [75]. *Chryseobacterium shandongense* has been isolated from soil, however its significance is still unknown beyond being a contaminant [76]. Scrub typhus, caused by *Orientia tsutsugamushi* infection, is a mite-borne febrile illness endemic in the Asia-Pacific region [77]. Common bacterial genera that are found in the human skin microbiome include *Micrococcus* and *Staphylococcus* [78]. *Burkholderia contaminans* is an emerging pathogen associated with cystic fibrosis and has been identified in sputum [78]. *Delftia acidovorans* is an aerobic, nonfermenting Gram-negative bacillus that is usually nonpathogenic environmental organism, which is rarely clinically significant [79]. Although *D. acidovorans* infection is usually associated with immunocompromised patients, there are reports documenting the infection in immunocompetent patients. The family *Corynebacteriaceae* is composed of the type genus *Corynebacterium* and many members of the family occupy diverse environments with some beneficial species, whereas other species are serious pathogens of humans and animals [80]. *Corynebacterium* is a Gram-positive bacterium whose manifestation of the infection depends on the specific host [81]. Contamination occurs through contact with infected animals and consumption of infected food, hence the isolation of the bacterium in raw beef mince, beef sausages, and beef patties. 

The genus *Staphylococcus* is a member of the family *Micrococcaceae*, which is a diverse group that have the ability to cause many diseases in humans and animals [82]. *Streptococcus pluranimalium* is a member of the *Streptococcus* genus that was isolated from diverse animal hosts and has been associated with subclinical mastitis, valvular endocarditis, and septicemia in animals [83]. Many *Campylobacter* species are naturally hosted by domesticated animals raised as food such as chicken, cattle, and pigs hence *Campylobacter hyointestinalis* has previously been isolated from the intestines of pigs with proliferative enteritis, feces of cattle and the intestine of a hamster [84]. *Campylobacter concisus* plays a role in the development of inflammatory bowel disease (IBD) whereas *Campylobacter sputorum* is primarily isolated from food animals such as cattle and sheep infrequently associated with human illness [85]. *Dichelobacter nodosus* (Dn) causes a debilitating and highly contagious disease called footrot in ruminants that results in necrotic hooves and significant economic losses in agriculture [86]. *Neisseria animaloris* is considered to be a commensal of the oral cavity of canine and feline and of public health importance as it is capable of causing systemic infections in animals and human beings [87]. Metagenomics revealed the presence of *N. animaloris* canine oral taxon 016 clone OB021 from raw beef mince. The highly thermos-resistant spore-forming bacteria in the group are a major threat in heat-treated foods as pasteurization heat may be insufficient in inactivating them [88,89]. Factors that may predict the composition of microbiota during processing and storage include environmental hygiene, composition, origin, huddle factors and conditions such as temperature, atmosphere, and pressure alongside the characteristics of most prevalent and resistant microorganisms [90]. 

### 4.2. Incidental Contamination Observed from the NCBI Nt Database 

The NGS technologies permits a much higher sensitivity and resolution. The taxonomy used can have significant effects on study results due to lack of consensus on the “best” reference database for taxonomic assignment of DNA sequences, however, for researchers interested in organisms from all domains, the NCBI nt database is a widely used reference as it contains comprehensive updated information for not only Bacteria and Archaea, but also Eukarya [91]. In numerous research areas, NGS-based approaches have been reported to be susceptible to contamination with undesired sequences (nontarget DNA) such as food web analysis [92], which highlights the functionality and power of sequencing-based approaches to identify organisms within the value chain without the need for culturing. The unusual incidental contamination in the samples from the analysis of sequences obtained from the NCBI nt database consisted of plant pathogens, bacteria, viruses, and fungi (*R. solanacearum*, *Enterobacteria*, *E. coli*, *Corynebacterium* spp., *Suttonella* spp., *M. bovoculi*, *S.* Typhimurium, *S.* Enteritidis, *S.* Weltevreden, *Streptococcus pluranimalium*, *Arthrobacter* spp., *B. paraconglomeratum*, *Mycobacterium smegmatis*, *S.* Agona, *Nocardioides* spp., *Sediminibacterium* spp., *S.* Gallinarum, African swine fever virus, *Fusarium* spp., *N. sphaerica*, *S. rostrate*, *Agaricaceae* spp., *S. commune*, *Curvularia* spp., *Bipolaris* spp., *Dothideomycetes* spp., and *Acremonium* spp.), which are discussed below.

*R. solanacearum* species are particularly destructive for vegetable crops, including potato, tomato, eggplant, and pepper plants and the species complex is responsible for bacterial wilt on a broad range of plant hosts comprising more than 200 species in at least 50 families [93,94] and is particularly destructive for vegetable crops, including potato, tomato, eggplant, and pepper plants [93,94]. The presence of *R. solanacearum* annotated sequences in beef sausages and rashers could be due to plant material added to the composite product.

PhiX174 belongs to the *Microviridae* family of bacteriophages and *Enterobacteria phage phiX174* is a single-stranded DNA (ssDNA) virus that infects *E. coli* [95]. Among the representative of the genetic diversity of the entire *E. coli* species, only 3% (8/291) of *E. coli* strains isolated from sewage, stools, drinking water, or the laboratory have been found to be sensitive to PhiX174 [95]. The presence of reads annotated as *Enterobacteria phage phiX174* isolate XC+Mad10im8 and *Enterobacteria* phage in biltong, mince, ham, and raw beef patties could have been due to pathogen reduction in the product and or production environment.

The identification of *E. coli*, *E. coli* plasmid pV003-c DNA, *E. coli* plasmid and *E. coli* plasmid pV044-c DNA reads in raw beef mince, mince, beef-pork-sausage and patties is a common occurrence in practice and was a common occurrence in this analysis irrespective of the database used. Animals and their environment are among the important sources of pathogenic *E. coli*, which may contaminate meat and meat products.

The genus *Suttonella* consists of Gram-negative, aerobic coccobacillus of *Cardiobacteriaceae* family and its natural habitat is the mucous membranes of the upper respiratory system [96]. The literature includes a limited number of case reports concerning fatal endocarditis in humans and birds due to infection in the extracardiac (respiratory system) and cardiac caused by the microorganism [97,98]. 

While the extent of host range, niche specialization, and genetic diversity of *M. bovoculi* is unknown, this bacterium is associated with infectious bovine keratoconjunctivitis (IBK) or “pinkeye” in cattle [98]. Beside its economic impact in livestock production due to IBK, it is of no zoonotic significance. There are no reports of its presence in beef mince, however, *M. bovoculi* strain 57,922 sequences in beef mince is of further research interest.

*S.* Typhimurium has previously been isolated in the USA [99] and similarly annotated reads were seen in beef mince, undefined mince and sausages. Furthermore, *S.* Enteritidis and *S.* Weltevreden sequence data was identified in raw mince, sausages, and patties. *S.* Agona strain 392869-2 has been reported to have originated from food factories at the time of a pan-European outbreak in 2008 with 163 confirmed cases reported [100]. Within the last years, *S.* Agona has been one of the top 20 most commonly reported serotypes causing human infections in the USA and based on the outbreak investigation results, there is evidence supporting the persistence of *Salmonella* over time in food processing facilities and highlights the need for consistent monitoring and control of organisms in the supply chain to minimize the risk of successive outbreaks [101]. The presence of *S.* Gallinarum str. 287/91 in non-chicken parties and sausage could be due to cross contamination. *Salmonella* Gallinarum biovar Gallinarum (*S.* Gallinarum) is one species specific poultry pathogen that causes major economic losses to the poultry industry worldwide. *S*. Gallinarum control relies mainly on the adoption of biosecurity programs, and success is dependent on accurate and fast detection [102]. 

*Streptococcus pluranimalium* is a recent member of the *Streptococcus* genus [103]. While the patho-biological properties of *S. pluranimalium* remain virtually unknown, *S. pluranimalium* has been described as a “promiscuous” pathogen in terms of its host and tissue tropism as it has been isolated from various tissues of multiple domestic animals and humans and the organism has not been previously reported to be present in raw beef parties [83]. 

*Arthrobacter* spp. was detected in beef patties. *Arthrobacter* are Gram-positive obligate aerobic bacteria that are usually found in soil and belong to the *Micrococcaceae* family [104]. The *Arthrobacter* are widespread in nature and their nutritional versatility enables them to inhabit diverse environments such as soil, sewage, and food [104,105]. The Arthrobacters have environmental and industrial relevance as some strains have applications in bioremediation and degradation of herbicides and pesticides from the environment [106,107]. 

The significance of *B. paraconglomeratum* sequences detected in raw beef patties is not known. *B. paraconglomeratum* are Gram-positive, facultative anaerobic bacteria [108]. Members of the genus *Brachybacterium* have been isolated from foods such as milk products and salt-fermented seafood as well as environmental samples and murine liver [109,110]. 

The transmission pattern and significance of the *M. smegmatis* strain RE001 detected in raw beef patties is not known. *M. smegmatis* may be found in lower animals, genital secretions of human beings, soil, dust, and water [111]. *Mycobacterium smegmatis* is recognized to be a human pathogen and human infections in skin or soft-tissue infections and in normal human-genital secretions are commonly related to immunosuppression [112]. 

There is limited information as to the role of *Nocardioides* spp., NCCP-1277 detected in the beef sausage. *Nocardioides* is a Gram-positive, mesophilic, and aerobic bacterial genus from the family *Nocardioidaceae* [113]. Bacteria classified in the genus *Nocardioides* are widely distributed in various environments such as soil, water, sediment, and sludge and no pathogenicity to plants or animals has been reported in any of its species [114]. They are often isolated as plant endophytes and are known to be capable of suppressing crop pathogens and as a tool in the safe bioremediation of a melamine-contaminated farmland [115,116,117].

The presence of *S. arietis* sequence data in beef sausage could be an indication of contaminated water used in production areas. In general, the main bacterial species associated with metal transformation in terrestrial and aquatic habitats include sulfate-reducing bacteria, sulfur-oxidizing bacteria, iron-oxidizing bacteria, and iron-reducing bacteria [117]. The impact of pure or artificially mixed culture bacteria on cast iron corrosion in water distribution pipelines has been studied [118]. Research studies have highlighted that corrosion-inducing bacteria include the IOB *Sediminibacterium* spp. [119]. *Sediminibacterium* genera which is isolated from sediment and activated sludge is highlighted to be one of the nitrate-dependent IOBs bacteria [120]. 

African swine fever virus (ASFV) is not a risk to human health and a closer analysis at the beef sausage where the African swine fever virus was isolated confirmed that the beef sausage was contaminated with *Sus scrofa* at 13%. ASF is a highly contagious hemorrhagic viral disease of domestic and wild pigs and causes major economic and production losses [121]. It is caused by *Asfarviridae* family, which are large DNA viruses that also infect ticks of the genus *Ornithodoros* [122]. 

The presence of sequences assigned to *Fusarium* spp. PVF24 internal transcribed spacer 1 in biltong, *Fusarium chlamydosporum* isolate Br-2 18S ribosomal RNA gene in raw mince, *Fusarium* spp. BAB-4621 18S ribosomal RNA gene in raw patties, *Fusarium* spp. TM1H51 internal transcribed spacer 1 in raw sausage and *Fusarium* spp. 1CD-3 internal transcribed spacer 1 in polonies could have been due to cross contamination. The genus *Fusarium* members are ubiquitous fungi frequently found in soils and plants and are a widely spread phytopathogens found in an extensive variety of hosts [123]. The genus species causes wilts and root rot disease, which produces secondary metabolites such as T2-toxin, zearalenone, and trichothecene, causing huge economic problems through losing crops, however, the genus *Fusarium* is seldom able to cause human infections [124]. 

The origin of *N. sphaerica* strain QY-6 18S ribosomal RNA gene sequences in raw mince and bacon could not be extrapolated as no studies have investigated *N. sphaerica* abundance in various food production environments in South Africa. *N. sphaerica* is an airborne filamentous fungus in the phylum *Ascomycota* that is found in soil, air, some cereal grains, and plants as a leaf pathogen [124]. Human infection by *N. sphaerica* have been reported among immunocompromised humans where the common response to *N. sphaerica* in humans is hay fever, human eye, and skin infections [125]. 

Segments of the *S. rostrata* strain 1296 18S ribosomal RNA gene was detected in raw patties. *S. rostrata* is a thermophilic fungus found in soils and a common plant pathogen, causing leaf spots as well as crown rot and root rot in grasses [126]. It is one of the species implicated uncommonly as opportunistic pathogens of humans where it is an etiologic agent of sinusitis, keratitis, and central nervous system vasculitis as well as cutaneous and subcutaneous mycoses [127]. 

The unexpected presence of segments belonging to the *Agaricaceae* spp. Am-13G 18S ribosomal RNA gene in raw patties could be due to cross contamination. The *Agaricaceae* are a family of basidiomycete fungi divided into four tribes distinguished largely by spore color [128]. Although many macrofungi including representatives from the Basidiomycota are edible and rich sources of important nutrients for humans, some are pathogenic to plants [129]. 

*S. commune* strain AZ1 18S ribosomal RNA gene sequences were detected in raw patties. *S. commune* is a mushroom-forming fungus, which has the ability to complete its life cycle in approximately 10 days and has worldwide distributions [130]. *S. commune* has the capability to secrete hydrolytic enzymes including xylanases and endoglucanases that are expressed in a wide range of substrates [131].

The presence of sequences assigned to *Curvularia* spp. GR1b 18S ribosomal RNA gene in sausage, *C. aeria* strain IP:+613.60 isolate ISHAMITS_ ID MITS1389 18S ribosomal RNA gene in sausage and *Curvularia* cf. *brachyspora* UFMGCB 6336 18S ribosomal RNA gene in ham could have been due to cross contamination. The genus *Curvularia* includes pathogens and saprobes of various plants, and opportunistic pathogens of humans and animals [132]. *Curvularia* spp. have been reported to be associated with air, aquatic environments, and soil [133]. *C. aeria* produces large, upright stroma and has been isolated from spot lesions on lettuce leaves [134]. Mycotic keratitis linked to *C. brachyspora* have been described in the context of some *Curvularia* spp. causing mycoses [135]. 

The presence of reads aligning to the *Bipolaris* spp. RM02 18S ribosomal RNA gene in sausage could have been due to the plant material added to the product. The genus *Bipolaris* consists of plant pathogens that are commonly associated with disease symptoms in diverse high value field crops [136,137]. 

The significance of sequences determined to originate from the uncultured endophytic fungus clone 51-01-58 18S ribosomal RNA gene in sausage, uncultured fungus clone FLITS03B04 internal transcribed spacer 1 in patties and uncultured fungus clone ASSC099 internal transcribed spacer 1 in polonies could not be established. Future attempts to culture these unknown fungal groups may provide key insights into the early evolution of fungi and their ecological and physiological significance in food environments. Endophytic fungi are ubiquitous and play an important role in the natural environment and occur within a wide range of hosts in diverse ecosystems [138]. 

The presence of segments annotated as *Dothideomycetes* spp. genotype css038 internal transcribed spacer 1 in ready-to-eat polony could be due to the plant material used for the formulation of the product. *Dothideomycetes* spp. is a phylogenetically diverse class within the fungal phylum, Ascomycota [139]. *Dothideomycetes* are known producers of toxins, especially plant host-specific toxins (HSTs) [140]. 

The presence of sequences assigned to the *Acremonium* spp. B28 internal transcribed spacer 1 in rashers could have been due to contaminated raw material and/or production environment. The genus *Acremonium* contains several species, which are mainly isolated from dead plant material and soil and recognized as opportunistic pathogens of immunocompromised man and animals [140,141]. *Acremonium* spp. are being increasingly recognized as opportunistic pathogens and it appears that the major predisposing factors comprises prolonged use of corticosteroids, splenectomy, and bone marrow transplantation [142]. Although rare, infections of humans by fungi of this genus have clinical manifestations that may include arthritis, osteomyelitis, peritonitis, endocarditis, pneumonia, cerebritis, and subcutaneous infection [143]. 

Taken together, the findings from this study that involved culture dependent methods targeting specific microorganisms and the culture-independent techniques that were applied to a smaller sample size illustrate the extent of microbial communities of meat on the South African market. Given this scenario, it is paramount to apply strict hygienic measures during processing of meat and meat products along the entire meat value chain from “farm to fork”. Furthermore, regular surveillance of indicator organisms and foodborne pathogens in the product and environment should be a crucial aspect for entities that are involved in handling and processing meat and meat products in addition to implementation and maintenance of Hazard Analysis Critical Control Point (HACCP). It is clear from the findings of this study that contamination of meat and meat products is complex and requires a multifaceted One Health approach at different levels by diverse stakeholders in order to protect the health of consumers.

## 5. Conclusions

This study represents the first comprehensive account of the simultaneous utilization of a combination of classical microbiological techniques and molecular techniques to evaluate the diversity of microorganisms that contaminate meat and products of animal origin placed on the South African domestic market. The data demonstrate diverse and highly variable microbial communities across meat and food of animal origin, which is important in the context of food safety, food labeling, biosecurity, and shelf life limiting spoilage organisms. The isolation of bacterial pathogens with zoonotic potential such as *Salmonella enterica* in foods highlights the necessity to tighten hygienic measures and enhance regular surveillance along the entire meat production system in order to curb the risk to consumers.

Overall, the findings of this study challenge the food industry to enhance interventions targeting specific FSO by the application of enhanced principles of Good Hygienic Practice (GHP) and Hazard Analysis Critical Control Point (HACCP) systems that are informed by microbiological empirical evidence. It provides the scientific basis that allows industry to select and implement risk based measures that control the hazard(s) of concern in a specific food or food operation and regulators to develop and implement effective inspection procedures to assess the adequacy of control measures implemented by industry. Future studies should focus on detailed characterization of the population structure of foodborne pathogens in order to understand their epidemiology, virulence, and antimicrobial resistance profiles.

## Figures and Tables

**Table 1:** The oligodeoxynucleotide sequences of the universal primers for mitochondrial 16S rRNA gene amplification. The letters in small case are Nextera adapter tails.

16S Forward	tcgtcggcagcgtcagatgtgtataagagacagGACGAGAAGACCCTATTGGAGC
16S Reverse	gtctcgtgggctcggagatgtgtataagagacagTCCGAGGTCRCCCCAACC

## Data Availability

Data is contained within the article or supplementary material.

## Supplementary Materials

The following are available online at https://www.mdpi.com/2076-2607/9/3/507/s1. Table S1: Number of reads (*n* ≥ 10) assigned to different genera obtained from various product types; Table S2. Summary of the distribution of meat and meat products that tested positive for *Campylobacter* species in all provinces of South Africa; Table S3: Proportion of meat and meat products that tested positive for *Bacillus cereus*; Table S4: Occurrence of *Clostridium perfringens* in meat and meat products from local and imported meat samples in South Africa; Table S5: Proportion of meat and meat products (South Africa and other countries) that tested positive for *Salmonella* species; Table S6: Summary of the distribution of meat and meat products that tested positive for *Yersinia enterocolitica* in all nine provinces of South Africa; Table S7: Summary of the proportion of meat and meat products that tested positive for *Staphylococcus aureus*.
