# Supplementary material for: Microbial Communities of Meat and Meat Products: An Exploratory Analysis of the Product Quality and Safety at Selected Enterprises in South Africa
Source: Microorganisms. 2021 Feb 27;9(3):507. doi: 10.3390/microorganisms9030507 (PMC7997435; doi:10.3390/microorganisms9030507)
Supplement: Supplementary file 1 [file microorganisms-09-00507-s001.pdf]

Table S1: Number of reads (n >= 10) assigned to different genera obtained from various product types

	Beef		Beef-biltong		Beef-mince		Beef-patties		Beef-sausage		Biltong		Mince		Patties		Sausage		
**Genus**	**kaiju**	**kraken**	**kaiju**	**kraken**	**kaiju**	**kraken**	**kaiju**	**kraken**	**kaiju**	**kraken**	**kaiju**	**kraken**	**kaiju**	**kraken**	**kaiju**	**kraken**	**kaiju**	**kraken**	**Total**
**Arcicella**	18	0	34	0	71	0	73	0	48	0	33	0	83	0	37	0	52	0	449
**Colwellia**	0	0	0	0	0	0	0	360	0	175	0	0	0	0	0	0	0	0	535
**Corynebacterium**	0	0	0	0	0	37	0	18	0	89	0	0	0	0	0	0	0	0	144
**Cutibacterium**	0	0	0	0	19	0	0	0	37	0	0	0	0	0	0	0	0	0	56
**Enterococcus**	0	0	0	0	0	0	0	0	11	0	0	0	0	0	0	0	0	0	11
**Fusobacterium**	0	0	0	0	0	0	0	0	13	12	0	0	0	0	0	0	0	0	25
**Klebsiella**	0	0	0	0	0	0	0	0	0	0	11	0	15	0	0	0	0	0	26
**Lactobacillus**	0	0	23	0	35	11	29	0	22	27	19	0	40	0	12	0	16	0	234
**Listeria**	0	0	0	0	0	0	0	0	27	0	0	0	0	0	0	0	0	0	27
**Mycobacterium**	0	0	0	0	0	0	0	0	19	0	0	0	0	0	0	0	0	0	19
**Mycobacteroides**	0	0	0	0	0	0	14	0	31	0	0	0	0	0	0	0	0	0	45
**Staphylococcus**	0	0	0	0	0	0	0	0	0	19	0	13	0	30	0	22	0	19	103
**Streptomyces**	0	0	0	0	0	0	0	0	12	0	0	0	0	0	0	0	0	0	12
**Suicoccus**	0	0	0	0	0	0	0	0	0	10	0	0	0	0	0	0	0	0	10
**Winogradskyella**	0	0	12	0	19	0	15	0	0	0	0	0	20	0	12	0	12	0	90
**Total**	18	0	69	0	144	48	131	378	220	332	63	13	158	30	61	22	80	19	1786

Table S2. Summary of the distribution of meat and meat products that tested positive for *Campylobacter* species in all provinces of South Africa.

		% of positive sample (n)	Provinces % (n)								
			**Eastern Cape**	**Free State**	**Gauteng**	**Kwa-Zulu Natal**	**Limpopo**	**Mpumalanga**	**North West**	**Northern Cape**	**Western cape**
**Meat type**	Raw-processed meat	14.38 (110/765)	16.67 (2/12)	8.11 (6/74)	29.71 (71/239)	1.41 (1/71)	7.89 (12/152)	9.26 (10/108)	9.43 (5/53)	5.56 (2/36)	5 (1/20)
	Ready to eat	2.29 (10/436)	0 (0/10)	0 (0/51)	0.70 (1/143)	4.0 (2/50)	7.35 (5/68)	2.22 (1/45)	0 (0/21)	3.57 (1/28)	0 (0/20)
	Raw-intact meat	7.00 (39/557)	3.51 (2/57)	1.9 (1/52)	7.89 (3/38)	18.18 (4/22)	8.33 (3/36)	6.78 (8/118)	12.31 (16/130)	3.64 (2/55)	0 (0/49)
**Food establishment**	Abattoir	5.13 (2/39)	0 (0/0)	0 (0/10)	11.11 (1/9)	0 (0/1)	0 (0/5)	20 (1/5)	0 (0/9)	0 (0/0)	0 (0/0)
	Butchery	5.98 (37/619)	5.71 (2/35)	3.45 (2/58)	10.79 (15/139)	6.9 (2/29)	8.47 (5/59)	8.11 (9/111)	1.64 (1/61)	1.41 (1/71)	0 (0/58)
	Processing plant	33.33 (20/60)	0 (0/2)	28.57 (2/7)	28.57 (6/21)	25 (2/8)	44.44 (8/18)	50 (2/4)	0 (0/0)	0 (0/0)	0 (0/0)
	Retail	9.62 (100/1040)*	4.76 (2/42)	2.94 (3/102)	21.12 (53/251)	2.86 (3/105)	4.02 (7/174)	4.64 (7/151)	14.92 (20/134)	8.33 (4/48)	3.13 (1/32)

**Species from which the food was derived**	Cattle	8.17 (88/1077)	5.88 (2/34)	1.80 (2/111)	18.52 (65/351)	0 (0/93)	4.79 (9/188)	3.79 (5/132)	3.03 (2/66)	3.70 (2/54)	2.08 (1/48)
	Game	9.09 (3/33)	0 (0/1)	0 (0/0)	0 (0/0)	0 (0/2)	6.67 (1/15)	22.22 (2/9)	0 (0/0)	0 (0/6)	0 (0/0)
	Sheep	0 (0/68)	0 (0/10)	0 (0/5)	0 (0/3)	0 (0/7)	0 (0/4)	0 (0/5)	0 (0/4)	0 (0/19)	0 (0/11)
	Mixed	24.44 (11/45)	0 (0/1)	25 (1/4)	30 (3/10)	0 (0/5)	44.44 (4/9)	50 (1/2)	9.09 (1/11)	50.0 (1/2)	0 (0/1)
	Pork	6.67 (9/135)	0 (0/11)	7.14 (1/14)	16.67 (4/24)	0 (0/11)	0 (0/22)	25 (4/16)	0 (0/12)	0 (0/14)	0 (0/11)
	Poultry	12 (48/400)	9.09 (2/22)	6.98 (3/43)	9.38 (3/32)	28 (7/25)	33.33 (6/18)	6.54 (7/107)	16.22 (18/111)	8.33 (2/24)	0 (0/18)
**Domestic meat samples**		**9.04 (159/1758)**	**5.06 (4/79)**	**3.95 (7/177)**	**17.86 (75/420)**	**4.90 (7/143)**	**7.81 (20/256)**	**7.01 (19/271)**	**10.29 (21/204)**	**4.20 (5/119)**	**1.12 (1/89)**
**Imported meat**	**POE**	**3.09 (8/259)**									

Table S3: Proportion of meat and meat products that tested positive for *Bacillus cereus*

		% of positive sample (n)	Provinces % (n)								
			**Eastern Cape**	**Free State**	**Gauteng**	**Kwa-Zulu Natal**	**Limpopo**	**Mpumalanga**	**North West**	**Northern Cape**	**Western cape**
**Meat type**	Raw-processed	7.19 (55/765)	16.67 (2/12)	5.40 (4/74)	7.53 (18/239)	5.63 (4/71)	7.24 (11/152)	7.69 (8/108)	3.77 (2/53)	8.33 (3/36)	15 (3/20)
	meat Ready to eat	2.75 (12/436)	10 (1/10)	1.96 (1/51)	2.10 (3/143)	6 (3/50)	1.47 (1/68)	2.22 (1/45)	0 (0/21)	3.57 (1/28)	0 (1/20)
	Raw-intact meat	2.15 (12/557)	3.51 (2/57)	1.92 (1/52)	10.53 (4/38)	9.09 (2/22)	2.78 (1/36)	0.85 (1/118)	0.77 (1/130)	0 (0/55)	0 (0/49)
**Food establishment**	Abattoir	5.13 (2/39)	0 (0/0)	0 (0/10)	22.22 (2/9)	0 (0/1)	0 (0/5)	0 (0/5)	0 (0/9)	0 (0/0)	0 (0/0)
	Butchery	3.07 (19/619)	5.71 (2/35)	1.72 (1/58)	3.6 (5/139)	3.45 (1/29)	3.39 (2/59)	2.70 (3/111)	1.64 (1/61)	1.41 (1/71)	5.17 (3/58)
	Processing plant	41.67 (25/60)	100 (2/2)	42.86 (3/7)	33.33 (7/21)	50 (4/8)	38.89 (7/18)	50 (2/4)	0 (0/0)	0 (0/0)	0 (0/0)
	Retail	3.18 (33/1039)	2.38 (1/42)	1.96 (2/102)	4.38 (11/251)	3.81 (4/105)	2.3 (4/174)	3.31 (5/151)	1.49 (2/134)	6.25 (3/48)	3.13 (1/32)

**Species from which the food was derived**	Cattle	4.7 (51/1077)	5.9 (2/34)	1.8 (2/111)	5.4 (19/351)	3.2 (3/93)	10.2 (9/188)	6.8 (9/132)	1.5 (1/66)	3.7 (2/54)	8.3 (4/48)
	Game	0 (0/33)	0 (0/1)	0 (0/0)	0 (0/0)	0 (0/2)	0 (0/15)	0 (0/9)	0 (0/0)	0 (0/6)	0 (0/0)
	Sheep	1.5 (1/68)	0 (0/10)	0 (0/5)	0 (0/3)	14.3 (1/7)	0 (0/4)	0 (0/5)	0 (0/4)	0 (0/19)	0 (0/11)
	Mixed	6.67 (3/45)	0 (0/1)	0 (0/4)	10.0 (1/10)	0 (0/5)	0 (0/9)	50 (1/2)	0 (0/11)	0 (1/2)	0 (0/1)
	Pork	12.6 (17/135)	18.2 (2/11)	28.6 (4/14)	54.2 (3/24)	27.3 (3/11)	18.2 (4/22)	0 (0/16)	0 (0/12)	0 (1/14)	0 (0/11)
	Poultry	1.8 (7/400)	4.5 (1/22)	0 (0/43)	15.6 (2/32)	8.0 (2/25)	0 (0/18)	0 (0/107)	1.8 (2/111)	0 (0/24)	0 (0/18)
**Domestic meat samples**		**4.5 (79/1758)**	**6.3 (5/79)**	**3.4 (6/177)**	**6 (25/420)**	**6.3 (9/143)**	**5.1 (13/256)**	**3.7 (10/271)**	**1.5 (3/204)**	**3.4 (4/119)**	**4.5 (4/89)**
**Imported meat**	**POE**	**2.7 (7/259)**									

Table S4: Occurrence of *Clostridium perfringens* in meat and meat products from local and imported meat samples in South Africa

		% of positive sample (n)	Provinces % (n)								
			**Eastern Cape**	**Free State**	**Gauteng**	**Kwa-Zulu Natal**	**Limpopo**	**Mpumalanga**	**North West**	**Northern Cape**	**Western cape**
**Meat type**	Raw-processed meat	23.53 (180/765)	33.33 (4/12)	10.81 (8/74)	37.66 (90/239)	5.63 (4/71)	22.37 (34/152)	18.52 (20/108)	20.75 (11/53)	13.89 (5/36)	20 (4/20)
	Ready to eat	11.24 (49/436)	0 (0/10)	3.92 (2/51)	20.98 (30/143)	14 (7/50)	4.41 (3/68)	8.89 (4/45)	4.76 (1/21)	0 (0/28)	10 (2/20)
	Raw-intact meat	23.52 (131/557)	5.26 (3/57)	5.77 (3/52)	44.74 (17/38)	22.73 (5/22)	22.22 (8/36)	32.20 (38/118)	41.54 (54/130)	0 (0/55)	6.12 (3/49)
**Food establishment**	Abattoir	51.28 (20/39)	0 (0/0)	40 (4/10)	55.56 (5/9)	0 (0/1)	40 (2/5)	80 (4/5)	55.56 (5/9)	0 (0/0)	0 (0/0)
	Butchery	15.19 (94/619)	14.29 (5/35)	5.17 (3/58)	32.37 (45/139)	20.69 (6/29)	11.86 (7/59)	7.21 (8/111)	32.79 (20/61)	0 (0/71)	0 (0/58)
	Processing plant	71.67 (43/60)	50 (1/2)	42.86 (3/7)	71.43 (15/21)	62.5 (5/8)	88.88 (16/18)	75 (3/4)	0 (0/0)	0 (0/0)	0 (0/0)
	Retail	19.15 (203/1039)	2.38 (1/42)	2.94 (3/102)	28.69 (72/251)	4.76 (5/105)	29.41 (20/174)	31.13 (47/151)	30.60 (41/134)	10.42 (5/48)	28.13 (9/32)

**Species from which the food was derived**	Cattle	24.1 (260/1077)	5.9 (2/34)	3.6 (4/111)	31.6 (111/351)	10.8 (10/93)	21.3 (40/188)	28.0 (37/132)	75.8 (50/66)	5.6 (3/54)	6.3 (3/48)
	Game	33.3 (11/33)	0 (0/1)	0 (0/0)	0 (0/0)	0 (0/2)	20 (3/15)	77.8 (7/9)	0 (0/0)	16.7 (1/6)	0 (0/0)
	Sheep	20.6 (14/68)	10 (1/10)	40 (2/5)	33.3 (1/3)	0 (0/7)	50 (2/4)	60 (3/5)	25.0 (1/4)	5.3 (1/19)	27.3 (3/11)
	Mixed	20 (9/45)	0 (0/1)	0 (0/4)	80.0 (8/10)	0 (0/5)	0 (0/9)	0 (0/2)	0 (0/11)	0 (0/2)	100 (1/1)
	Pork	13.3 (18/135)	0 (0/11)	14.3 (2/14)	33.3 (8/24)	27.3 (3/11)	0 (0/22)	12.5 (2/16)	25 (3/12)	0 (0/14)	0 (0/11)
	Poultry	12 (48/400)	18.2 (4/22)	11.6 (5/43)	28.1 (9/32)	12 (3/25)	0 (0/18)	12.1 (13/107)	10.8 (12/111)	0 (0/24)	0 (2/18)
**Domestic meat samples**		**20.5 (360/1758)**	**8.9 (7/79)**	**7.3 (13/177)**	**32.6 (137/420)**	**11.2 (16/143)**	**17.6 (45/256)**	**22.9 (62/271)**	**32.4 (66/204)**	**4.2 (5/119)**	**10.1 (9/89)**
**Imported meat**	**POE**	**19.3(50/259)**									

TableS5: Proportion of meat and meat products (South Africa and other countries) that tested positive for *Salmonella* species

Samples type		% of positive sample (n)	Provinces % (n)								
			**Eastern Cape**	**Free State**	**Gauteng**	**Kwa-Zulu Natal**	**Limpopo**	**Mpumalanga**	**North West**	**Northern Cape**	**Western Cape**
**Meat type**	Raw-processed	3.9 (30/765)	8.3 (1/12)	3.8 (3/74)	3.4 (8/239)	1.4 (1/71)	8.6 (13/152)	2.8 (3/108)	0 (0/53)	2.8 (1/36)	0 (0/20)
	Ready to eat	2.8 (12/436)	0 (0/10)	0 (0/51)	2.8 (4/143)	6 (3/50)	4.4 (3/68)	2.2 (1/45)	0 (0/21)	3.6 (1/28)	0 (0/20)
	Raw-intact meat	1.8 (10/557)	3.5 (2/57)	1.9 (1/52)	2.6 (1/38)	0.1 (1/22)	0 (0/36)	0 (0/118)	2.3 (3/130)	1.8 (1/55)	2.0 (1/49)
**Food establishment**	Abattoir	0 (0/39)	0 (0/0)	0 (0/10)	0 (0/9)	0 (0/1)	0 (0/5)	0 (0/5)	0 (0/9)	0 (0/0)	0 (0/0)
	Butchery	3.4 (21/619)	5.7 (2/35)	3.5 (2/58)	2.2 (3/139)	6.9 (2/29)	11.9 (7/59)	0.9 (1/111)	1.6 (1/61)	1.4 (1/71)	1.8 (1/58)
	Processing plant	6.7 (4/60)	0 (0/2)	14.3 (1/7)	5.0 (1/21)	25 (2/8)	0 (0/18)	0 (0/4)	0 (0/0)	0 (0/0)	0 (0/0)
	Retail	2.8 (29/1039)	2.4 (1/42)	0.98 (1/102)	3.6 (9/251)	0.95 (1/105)	5.1 (9/174)	2.0 (3/151)	1.5 (2/134)	4.2 (2/48)	0 (/32)

**Species from which the food was derived**	Cattle	3.2 (34/1077)	2.9 (1/34)	2.7 (3/111)	3.4 (12/351)	1.1 (1/93)	6.4 (12/188)	3.0 (4/132)	0 (0/66)	1.9 (1/54)	0 (0/48)
	Game	12.1 (4/33)	0 (0/1)	0 (0/0)	0 (0/0)	0 (0/2)	13.3 (2/15)	0 (0/9)	0 (0/0)	16.7 (1/6)	0 (0/0)
	Sheep	(0/68)	0 (0/10)	0 (0/5)	0 (0/3)	0 (0/7)	0 (0/4)	0 (0/5)	0 (0/4)	0 (0/19)	0 (0/11)
	Mixed	2.2 (1/45)	0 (0/1)	(0/4)	0 (0/10)	0 (0/5)	11.1 (1/9)	0 (/2)	0 (0/11)	0 (0/2)	0 (0/1)
	Pork	1.5 (2/135)	0 (/11)	0 (0/14)	9.1 (1/24)	0 (0/11)	4.6 (1/22)	0 (/16)	0 (0/12)	0 (0/14)	0 (0/11)
	Poultry	2.8 (11/400)	9.1 (2/22)	2.4 (1/43)	0 (0/32)	4.0 (1/25)	0 (0/18)	0 (/107)	2.7 (3/111)	4.2 (1/24)	5.6 (1/18)
**Domestic meat samples**		**2.9 (51/1758)**	**3.8 (3/79)**	**2.3 (4/177)**	**3.1 (13/420)**	**3.5 (5/143)**	**6.3 (16/256)**	**1.5 (4/271)**	**1.5 (3/204)**	**2.5 (3/119)**	**1.1 (1/89)**
**Imported meat**	**POE**	**5.0(13/259)**									

Table S6: Summary of the distribution of meat and meat products that tested positive for *Yersinia enterocolitica* in all nine provinces of South Africa

		% of positive sample (n)	Provinces % (n)								
			**Eastern Cape**	**Free State**	**Gauteng**	**Kwa-Zulu Natal**	**Limpopo**	**Mpumalanga**	**North West**	**Northern Cape**	**Western cape**
**Meat type**	Raw-processed meat	30.07 (230/765)	33.33 (4/12)	27.03 (20/74)	33.89 (81/239)	19.72 (14/71)	25 (38/152)	31.48 (34/108)	35.85 (19/53)	38.89 (14/36)	30 (6/20)
	Ready to eat	2.83 (13/436)	0 (0/10)	0 (0/51)	4.9 (7/143)	4.0 (2/50)	1.47 (1/68)	2.22 (1/45)	0 (0/21)	3.57 (1/28)	0 (1/20)
	Raw-intact meat	29.98 (167/557)	33.33 (19/57)	28.84 (15/52)	31.59 (12/38)	36.36 (8/22)	36.11 (13/36)	29.66 (35/118)	23.08 (30/130)	36.36 (20/55)	30.61 (15/49)
**Food establishment**	Abattoir	35.90 (14/39)	0 (0/0)	30 (3/10)	44.44 (4/9)	0 (0/1)	40 (2/5)	40 (2/5)	33.33 (3/9)	0 (0/0)	0 (0/0)
	Butchery	22.78 (141/619)	28.57 (10/35)	17.24 (10/58)	34.53 (48/139)	24.14 (7/29)	25.42 (15/59)	17.12 (19/111)	16.39 (10/61)	16.90 (12/71)	17.24 (10/58)
	Processing plant	35 (21/60)	100 (2/2)	28.57 (2/7)	28.57 (6/21)	37.5 (3/8)	38.89 (7/18)	25 (1/4)	0 (0/0)	0 (0/0)	0 (0/0)
	Retail	21.17 (234/1039)	26.19 (11/42)	19.61 (20/102)	16.73 (42/251)	13.33 (14/105)	16.09 (28/174)	31.79 (48/151)	26.87 (36/134)	47.91 (23/48)	37.5 (12/32)

Table S7: Summary of the proportion of meat and meat products that tested positive for *Staphylococcus aureus*

		% of positive sample (n)	Provinces % (n)								
			Eastern Cape	Free State	Gauteng	Kwa-Zulu Natal	Limpopo	Mpumalanga	North West	Northern Cape	Western cape
**Meat type**	Raw-processed meat	72.55 (555/765)	75 (9/12)	71.62 (53/74)	80.75 (193/239)	77.46 (55/71)	72.37 (110/152)	60.19 (65/108)	64.15 (34/53)	63.89 (23/36)	65 (13/20)
	Ready to eat	33.26 (145/436)	30 (3/10)	23.53 (12/51)	37.06 (53/143)	32 (16/50)	36.76 (25/68)	37.78 (17/45)	33.33 (7/21)	25 (7/28)	25 (5/20)
	Raw-intact meat	71.81 (400/557)	75.44 (43/57)	76.92 (40/52)	81.58 (31/38)	72.72 (16/22)	80.56 (29/36)	66.10 (78/118)	69.23 (90/130)	63.63 (35/55)	77.55 (38/49)
**Food establishment**	Abattoir	38.46 (15/39)	0 (0/0)	70 (7/10)	44.44 (4/9)	0 (0/1)	20 (1/5)	20 (1/5)	22.22 (2/9)	0 (0/0)	0 (0/0)
	Butchery	63 (390/619)	74.29 (26/35)	70.69 (41/58)	57.55 (80/139)	79.31 (23/29)	59.32 (49/59)	45.95 (51/111)	57.37 (35/61)	53.52 (38/71)	81.03 (47/58)
	Processing plant	83.33 (50/60)	100 (2/2)	71.43 (5/7)	80.95 (17/21)	75 (6/8)	94 (17/18)	75 (3/4)	0 (0/0)	0 (0/0)	0 (0/0)
	Retail	62.08 (645/1039)	64.29 (27/42)	50.98 (52/102)	70.12 (176/251)	55.23 (58/105)	55.74 (97/174)	69.53 (105/151)	70.15 (94/134)	56.25 (27/48)	28.13 (9/32)
